# XRCC1 mediated the development of cervival cancer through a novel Sp1/Krox-20 swich

**DOI:** 10.18632/oncotarget.21040

**Published:** 2017-09-16

**Authors:** Qingtao Meng, Shizhi Wang, Weiyan Tang, Shenshen Wu, Na Gao, Chengcheng Zhang, Xiaoli Cao, Xiaobo Li, Zhengdong Zhang, Michael Aschner, Hua Jin, Yue Huang, Rui Chen

**Affiliations:** ^1^ Key Laboratory of Environmental Medicine Engineering, Ministry of Education, School of Public Health, Southeast University, Nanjing, China; ^2^ Medical Oncology, Jiangsu Cancer Hospital, Nanjing, China; ^3^ Institute of Bioinformatics, Heinrich Heine University, Düsseldorf, Germany; ^4^ Clinical Lab, Nantong Tumor Hospital, Nantong, China; ^5^ Department of Environmental Genomics, Jiangsu Key Laboratory of Cancer Biomarkers, Prevention and Treatment, Cancer Center, Nanjing Medical University, Nanjing, China; ^6^ Department of Molecular Pharmacology, Albert Einstein College of Medicine, Bronx, NY, USA; ^7^ Core Laboratory, Nantong Tumor Hospital, Nantong, China; ^8^ Department of Pathology, Nanjing First Hospital, Nanjing Medical University, Nanjing, China; ^9^ State Key Laboratory of Respiratory Disease, Institute for Chemical Carcinogenesis, Guangzhou Medical University, Guangzhou, China

**Keywords:** base excision repair, XRCC1, cervical cancer, Krox-20, specificity protein 1

## Abstract

Cervical cancer is the second leading cause of mortality among women. Impairment of the base excision repair (BER) pathway is one of the major causes of the initiation and progression of cervical cancer. However, whether the polymorphisms of the BER pathway components (i.e., *HOGG1, XRCC1, ADPRT*, and *APE1*) can affect the risk of cervical cancer remains unknown. Herein, we applied a hospital-based case-control study covering two independent cohorts and a subsequent functional assay to determine the roles of the single nucleotide polymorphisms (SNPs) of the BER pathway genes in cervical cancer. Results indicated that the *XRCC1* rs3213245 (-77TC) TT genotype was associated with an increased risk of cervical cancer. The immunohistochemistry assay showed that XRCC1 protein expression levels were upregulated in cervical cancer patients with the *XRCC1* rs3213245 CC genotype compared with the CT or TT genotypes. Further, results from ChIP assay showed that Sp1 could bind to the −77 site and that the rs3213245 C genotype promoted the binding of Sp1 to the *XRCC1* promoter. Moreover, ChIP/Re-ChIP assays revealed that transcription factor Krox-20 was recruited to the XRCC1 rs3213245 mutation region and regulated the transcription of the *XRCC1* gene by interacting with Sp1, ultimately mediated cervical cancer development. In summary, the findings indicated that the functional *XRCC1* SNP rs3213245 was associated with the risk of cervical cancer based on the Sp1/Krox-20 switch.

## INTRODUCTION

Cervical cancer is the second most common malignant tumor found in women worldwide accounting for 7.5% of all female cancer deaths [1]. The base excision repair (BER) pathway is responsible for repairing the DNA single-strand breaks caused by oxidative stress (such as reactive oxygen species, ROS), thereby maintaining genomic integrity. Impaired BER pathways are considered the major cause of cervical cancer [2]. However, it remains unknown whether the genetic polymorphisms of the key modulators in the BER pathway affect cervical cancer susceptibility.

The apurinic/apyrimidinic endonuclease 1 (*APE1*), adenosine diphosphate ribosyltransferase (*ADPRT*), human oxoguanine glycosylase1 (*HOGG1*) and X-ray cross complementing group 1 (*XRCC1*) are important regulators of the BER pathway. The abasic (AP) site aroused by DNA single-strand breaks can be recognized and excised by *APE1*. The association between *APE1* rs1760944 (−656T>G) and rs1130409 (Asp148Glu) polymorphisms and the risk of renal cell carcinoma and breast cancer have been indicated in several case-control studies [3, 4]. HOGG1 removes 8-oxoG from the damaged DNA for further repair [5]. However, the HOGG1 (rs1052133) Ser genotype has failed to repair broken DNA and has been demonstrated to increase cancer susceptibility in the gallbladder cancer [6] and renal cell carcinoma [7]. Few studies have focused on the *HOGG1* genetic variants and cervical cancer susceptibility. XRCC1 serves as the scaffold protein in the BER pathway, which recognizes DNA breaks and interacts with DNA polymerase β, DNA ligase III, and other components to repair Single-Strand Breaks (SSBs) [8]. Many studies have demonstrated that *XRCC1* polymorphisms (i.e., rs1799782, Arg194Trp; rs25489, Arg280His; and rs25487, Arg399Gln) are associated with increased risk of cancer [9, 10]. The rs3213245 polymorphism located in the *XRCC1* promoter is associated with the enhanced risk of breast and lung cancer [11, 12]. Moreover, rs3213245 (-77TC) alters the affinity of several transcription factors binding to the promoter, such as specificity protein 1 (Sp1) [13]. Sp1, which is one of the best-characterized transcriptional activators [14], plays a prominent role in cell-cycle development and regulation [15–18].

In the present study, case-control studies were conducted to analyze the association between the polymorphisms of BER components and the risk of cervical cancer in a Chinese population. Further, ChIP/Re-ChIP and IHC assays were also performed to evaluate the roles of these SNPs in cervical cancer.

## RESULTS

### Demographic and clinical characteristics of the study subjects

The clinical characteristics of the enrolled testing and validation cohorts were evaluated (Table 1). No significant differences in age distribution (*P* = 0.726) or abortion (*P* = 0.565) were detected between cases and controls in the testing cohort. However, more cases with higher parity (≥2) (41.7%) and premenopausal status (44.0%) were observed. Moreover, most of the cases with cervical cancer, have been diagnosed as squamous-cell carcinoma (94.2%) at the first stage of invasion (68.0%). The demographic and clinical characteristics of the subjects in the validation cohort were almost the same with those in the testing cohort (Table 1).

**Table 1:** Frequency distribution of select characteristics in cervical cases and controls

Variables^a^	Test set		*P*	Validation set		*P*
	Cases	Controls		Cases	Controls	
	n = 571 (%)	n = 657 (%)		n = 608 (%)	n =1165 (%)	
Age, year (mean ± SD)	47.5 ± 10.1	47.3 ± 10.6	0.726	51.5±9.4	51.6±10.7	0.971
Parity						
0-1	324 (58.3)	446 (75.2)	< 0.001	323(54.2)	800(72.6)	<0.001
≥ 2	232 (41.7)	147 (24.8)		273(45.8)	302(27.4)	
Abortion						
No	377 (70.5)	402 (72.0)	0.565	242(40.7)	454(41.5)	0.762
Yes	158 (29.5)	156 (28.0)		352(59.3)	640(58.5)	
Menopausal status						
Premenopausal	240 (44.0)	127 (21.1)	< 0.001	318(52.65)	436(39.7)	<0.001
Postmenopausal	306 (56.0)	476 (78.9)		286(47.35)	661(60.3)	
Histologic types						
Squamous cell carcinoma	538 (94.2)			550(90.5)		
Adenocarcinomas	24 (4.2)			31(5.1)		
Adenosquamous carcinoma	4 (0.7)			16(2.6)		
Others^b^	5 (0.9)			11(1.8)		
Stage						
I	383 (68.0)			391(64.3)		
II	145 (25.8)			209(34.4)		
III	28 (5.0)			2(0.3)		
IV	7 (1.2)			6(1.0)		

^a^ Some cases lack information of selected variables.

^b^ Other histological types, such as cervical choriocarcinoma.

### HOGG1, XRCC1, ADPRT, and APE1 polymorphisms and risk of cervical cancer

The genetic variants of the key components (i.e., *HOGG1, XRCC1, ADPRT*, and *APE1*) in the BER pathway and the risk of cervical cancer in the testing cohort were analyzed. The genotypic frequencies of the selected polymorphisms did not deviate significantly from Hardy-Weinberg values in either test cases or controls (Table 2, *P* > 0.05 for the combined set). The results showed that the *XRCC1* (rs3213245) TT genotype increased the risk of cervical cancer (adjusted OR = 0.63; 95% CI = 0.46–0.86 for the testing set; adjusted OR = 0.57; 95% CI = 0.44–0.73 for the validation set; and adjusted OR = 0.62, 95% CI = 0.51–0.75 for the combined set, TT genotype reference). However, no significant associations were observed between the *HOGG1* (rs1052133, Ser326Cys), *XRCC1* (rs1799782, Arg194Trp; rs25487, Arg399Gln; rs25489, Arg280His), *ADPRT* (rs1136410, Val762Ala), or *APE1* (rs1130409, Asp148Glu; rs1760944, -656TG) polymorphisms and the risk of cervical cancer (Table 2).

**Table 2:** Association between genetic polymorphisms in the base excision repair genes and cervical cancer risk

Genotype		Test set			Validation set			Combined set		
		Cases/controls	P	Adjusted OR	Cases/controls	P	Adjusted OR	Cases/controls	P	Adjusted OR
rs3213245	TT	462/501	0.0357		493/841	0.0002		955/1342	<.0001	
	TC	104/140		0.69(0.50-0.96)	107/293		0.58(0.45-0.76)	211/433		0.65(0.54-0.80)
	CC	5/16		0.13(0.03-0.49)	8/31		0.43(0.19-0.97)	13/47		0.31(0.16-0.62)
	TC/CC	109/156	0.048	0.63(0.46-0.86)	115/324	<.0001	0.57(0.44-0.73)	224/480	<.0001	0.62(0.51-0.75)
	C allele	0.0998/0.1309			0.1012/0.1524			0.1005/0.1446		
	HWE	0.7479/0.1039			0.4274/0.3695			0.7256/0.0922		
rs1052133	GG	196/228	0.3279		216/414	0.9103		412/642	0.7961	
	GC	276/335		1.00(0.75-1.32)	301/568		1.05(0.83-1.32)	577/903		1.02(0.86-1.21)
	CC	99/94		1.36(0.92-1.99)	91/183		0.95(0.69-1.31)	190/277		1.07(0.85-1.35)
	C allele	0.4151/0.3980			0.3972/0.4009			0.4059/0.3998		
	HWE	0.9135/0.1006			0.4042/0.6083			0.6119/0.1633		
rs1760944	AA	182/211	0.976		199/386	0.9715		381/597	0.9301	
	AC	285/324		1.01(0.75-1.34)	298/564		1.06(0.84-1.35)	583/888		1.02(0.86-1.22)
	CC	104/122		1.06(0.73-1.54)	111/215		1.04(0.77-1.41)	215/337		1.00(0.80-1.26)
	C allele	0.4317/0.4323			0.4276/0.4266			0.4296/0.4286		
	HWE	0.6805/0.9033			0.9757/0.7216			0.7579/0.8315		
rs1130409	TT	182/192	0.6014		191/350	0.7823		373/542	0.5295	
	TG	282/338		0.95(0.71-1.26)	304/586		0.93(0.73-1.18)	586/924		0.93(0.78-1.11)
	GG	107/127		0.96(0.66-1.38)	113/229		0.94(0.69-1.28)	220/356		0.92(0.73-1.15)
	G allele	0.4343/0.4505			0.4359/0.4481			0.4351/0.4490		
	HWE	0.9034/0.3164			0.6799/0.5622			0.7034/0.2869		
rs1136410	TT	188/192	0.2215		254/425	0.0898		442/617	0.087	
	TC	280/325		0.92(0.69-1.23)	256/529		0.76(0.60-0.95)	536/854		0.85(0.72-1.01)
	CC	103/140		0.71(0.49-1.03)	98/211		0.78(0.57-1.05)	201/351		0.78(0.62-0.98)
	C allele	0.4256/0.4604			0.3717/0.4082			0.3978/0.4270		
	HWE	0.9436/0.9098			0.0151/0.0401			0.0793/0.0720		
rs1799782	CC	270/287	0.1799		286/585	0.4467		556/872	0.685	
	CT	240/310		0.81(0.62-1.05)	257/463		1.09(0.87-1.36)	497/773		0.97(0.82-1.14)
	TT	61/60		1.05(0.68-1.64)	65/117		1.18(0.82-1.68)	126/177		1.11(0.85-1.45)
	T allele	0.317/0.3272			0.3183/0.2991			0.3176/0.3093		
	HWE	0.4835/0.0664			0.523/0.0748			0.344/0.7652		
rs25489	GG	461/526	0.8963		492/927	0.7778		953/1453	0.6884	
	GA	102/123		1.03(0.74-1.43)	109/225		0.87(0.66-1.14)	211/348		0.96(0.79-1.18)
	AA	8/8		1.25(0.41-3.85)	7/13		1.20(0.45-3.16)	15/21		1.15(0.57-2.33)
	A allele	0.1033/0.1058			0.1012/0.1077			0.1022/0.1070		
	HWE	0.3899/0.7893			0.7282/0.8742			0.3942/0.9746		
rs25487	GG	316/374	0.8493		333/648	0.7711		649/1022	0.7375	
	GA	218/243		1.07(0.82-1.40)	233/429		1.11(0.89-1.38)	451/672		1.08(0.92-1.27)
	AA	37/40		0.98(0.57-1.68)	42/88		0.91(0.60-1.38)	79/128		0.97(0.71-1.33)
	A allele	0.1033/0.1058			0.1012/0.1077			0.1022/0.1070		
	HWE	0.3899/0.7893			0.7282/0.8742			0.3942/0.9746		

^a^Adjusted for age, parity, and menopausal status in the logistic regression model.

The associations between these genetic variants and the risk of cervical cancer were further evaluated by stratification analysis. As showed in Table 3, the *XRCC1* (rs3213245) TT genotype increased the risk of cervical cancer in the subgroups of aged >49 years (adjusted OR = 0.47, 95% CI = 0.36–0.62, TT genotype reference). We also observed a similar result in each subgroups of parity, abortion, and premenopausal status. Moreover, a significantly increased risk was also found in the early stage of cervical cancer (adjusted OR = 0.63, 95% CI = 0.52–0.76, TT genotype reference).

**Table 3:** Stratified analysis of XRCC1 rs3213245 genotypes associated with cervical cancer risk by the selected variables

Variables	Genotypes (cases/controls)		*P*	Adjusted OR (95% CI)^a^
	TT	TC/CC		
Age (years)				
≤49	503/696	129/205	0.2744	0.82 (0.63-1.07)
> 49	452/646	95/275	<.0001	0.47 (0.36-0.62)
Parity				
0-1	522/915	125/331	0.0005	0.68 (0.54-0.87)
≥ 2	412/322	93/127	0.0003	0.53 (0.39-0.73)
Abortion				
No	330/439	70/169	0.0002	0.51 (0.37-0.72)
Yes	588/773	141/271	0.0012	0.65 (0.51-0.83)
Menopausal status				
Premenopausal	505/679	119/233	0.003	0.69 (0.53-0.90)
Postmenopausal	429/567	97/221	<.0001	0.55 (0.42-0.73)
Stage				
I/II	912/1342	216/480	<.0001	0.63 (0.52-0.76)
III/IV	30/1342	7/480	0.3092	0.54 (0.24-1.23)

^a^Adjusted for age, parity, and menopausal status in the logistic regression model.

### XRCC1 rs3213245 TT genotype decreased XRCC1 protein expression through effecting the binding affinity of Sp1/Krox-20 to the XRCC1 promoter

**Figure 1:**
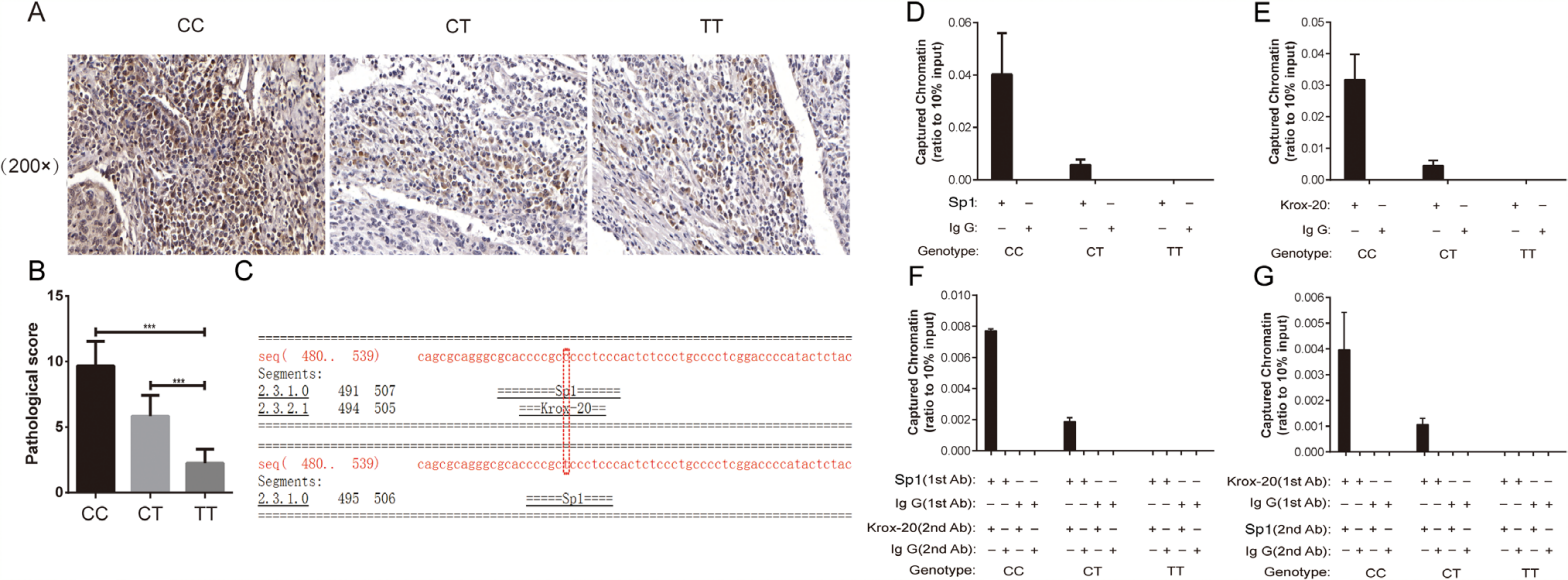
XRCC1 rs3213245 CC genotype promoted tumoral XRCC1 expression in patients IHC assay reveals that massive XRCC1-positive cells are observed in sections from cervical cancer patients carrying the XRCC1 rs3213245 CC genotype. **(A)** Representative IHC images and **(B)** IHC staining scores are shown. *P < 0.05. **(C)** XRCC1 −77 site located in the Sp1 binding motif according to the Alibaba2 Bioinformatics database. **(D)** A Sp1 ChIP assay was performed with different XRCC1 rs3213245 genotypes in cervical cancer patients. **(E)** Krox-20 ChIP assay was performed with different XRCC1 rs3213245 genotypes in cervical cancer patients. **(F** and **G)** Re-ChIP experiments were performed on formaldehyde-crosslinked DNA prepared from the human peripheral white blood cells of cervical cancer patients. For all genotypes, the chromatin–Sp1 complex was re-immunoprecipitated using anti-human Krox-20 (F), or the chromatin–Krox-20 complex was re-immunoprecipitated using anti-human Sp1 (G). The antibodies were used as indicated. Equal amounts of input and immunoprecipitated DNA were quantified using real-time quantitative PCR.

To further determine whether the *XRCC1* rs3213245 polymorphism can affect its protein expression, a total of 60 paraffin-embedded tissue sections were selected for immunohistochemistry (IHC) analysis. The frequency distributions of the XRCC1 rs3213245 CC, CT, and TT genotypes were 12, 24, and 24, respectively. The IHC assay showed that the XRCC1 protein expression levels were upregulated in cervical cancer patients with the *XRCC1* rs3213245 CC genotype compared with the CT or TT genotypes according to the staining scores (Figure 1A and 1B).

The XRCC1 rs3213245 SNP was located at the −77 promoter site which was contained in the Sp1 binding motif according to the Alibaba2 Bioinformatics database (Figure 1C). It is here hypothesized that the genetic variant might alter the XRCC1 transcription factor affinity on the Sp1 binding site. In this case, the mechanisms of action were determined and the ability of Sp1 to bind to the −77 site with ChIP analysis was assessed. Finally, a total of 9 cervical cancer patients with three different combined genotypes was selected to perform the ChIP and Re-ChIP experiments. Results indicated that the rs3213245 C genotype dramatically promoted Sp1 binding to the XRCC1 promoter, but the TT genotype did not (Figure 1D). Inspired by the Alibaba2 Bioinformatics database, we then proposed that Krox-20, one transcription factor overlapped with Sp1, might be regulated by this SNP. The following *in vivo* ChIP assay further evaluated this hypothesis (Figure 1E). Thus, we speculated that Sp1 and Krox-20 might co-regulate the XRCC1 transcription. We next performed sequential ChIP (Re-ChIP) assay to verify this hypothesis. Interestingly, we found that the XRCC1 rs3213245 SNP might affect the binding ability of Sp1 and Krox-20 complex to the chromatin (Figure 1F-1G).

### Sp1 regulated the transcription of the XRCC1 by recruiting Krox-20 to the rs3231245 mutation region

**Figure 2:**
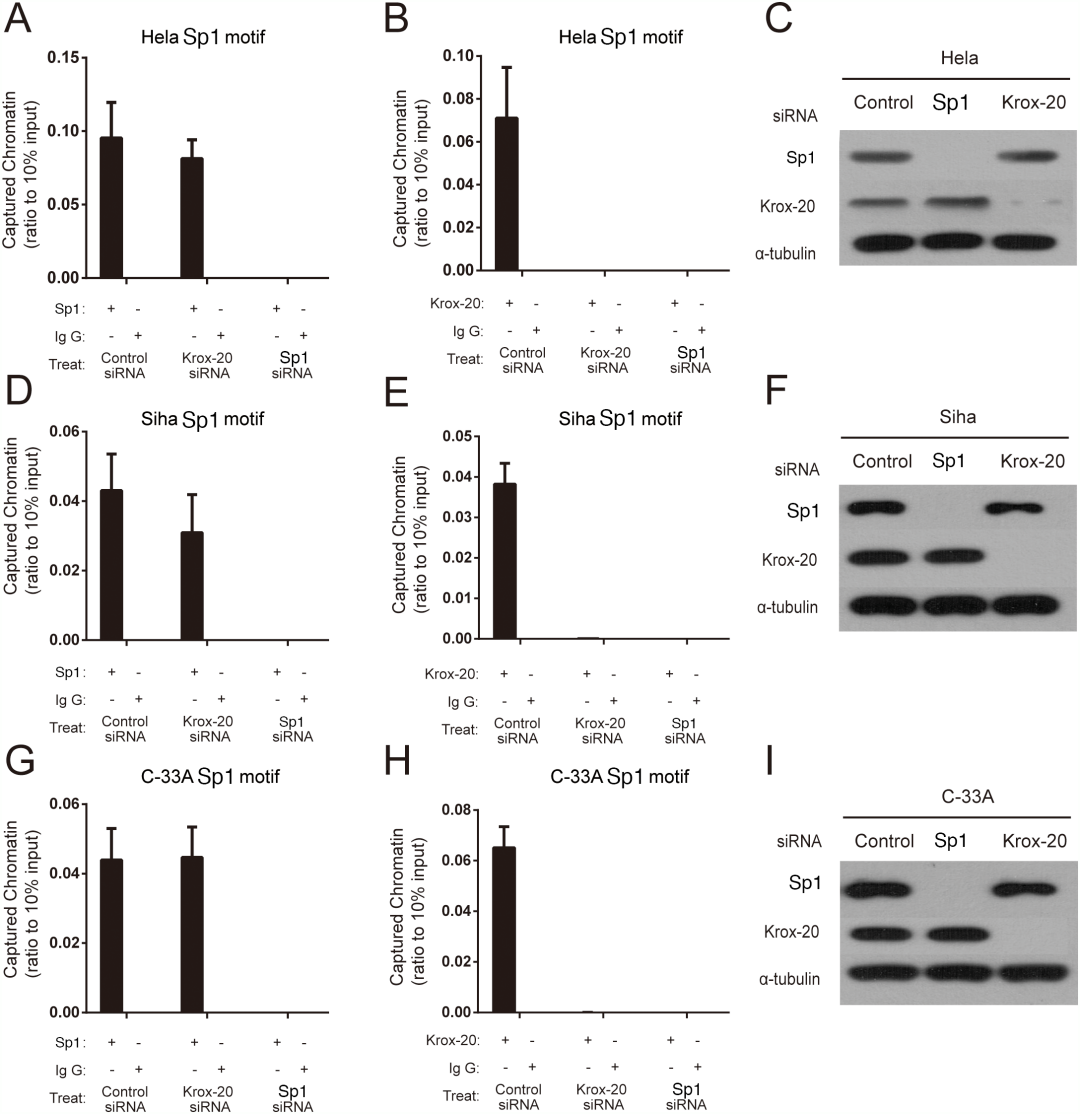
Sp1 regulated the transcription of the XRCC1 by recruiting Krox-20 to the rs3231245 mutation region **(A, B, D, E, G** and **H)** Binding affinity of Sp1 and Krox-20 to chromatin. **(C, F** and **I)** Confirmation of protein expression of Sp1 and Krox-20 in siRNA-transfected cells by Western blot analysis.

The siRNA knockdown experiments were then used to explore the manner of Sp1 and Krox-20 binding to the *XRCC1* promoter region in HeLa (HPV18–positive), SiHa (HPV16–positive), and C–33A (HPV–negative) cells. We found that Sp1 might bind with the rs3213245 mutation region when transfected with negative control or Krox-20 siRNA, rather than Sp1 siRNA (Figure 2A, 2D, 2G). However, Krox-20 failed to bind with the rs3213245 mutation region when transfected with Sp1 siRNA (Figure 2B, 2E, 2H). The knockdown was confirmed by Western blot (Figure 2C, 2F and 2I). In addition, the XRCC1 mRNA level of cervical cancer patients was determined to verify the transcriptional regulation of XRCC1 by the Sp1-Krox-20 complex *in vivo*. The frequency distribution of CC, CT, and TT was 2, 18, and 20, respectively. Results showed that individuals with CT/TT genotypes at the rs3213245 had significantly lower XRCC1 levels than those with the CC genotype (Supplementary Figure 1).

## DISCUSSION

Cervical cancer is still the leading cause of cancer-related deaths among women. Dysfunctions of DNA repair pathways, particularly the BER pathway, have also been considered one of the major causes of cancer [2]. However, few studies have focused on the association between the functional polymorphisms in the BER pathway and cervical cancer susceptibility. The present study demonstrated that genetic variants in the BER pathway are associated with the risk of cervical cancer.

**Figure 3:**
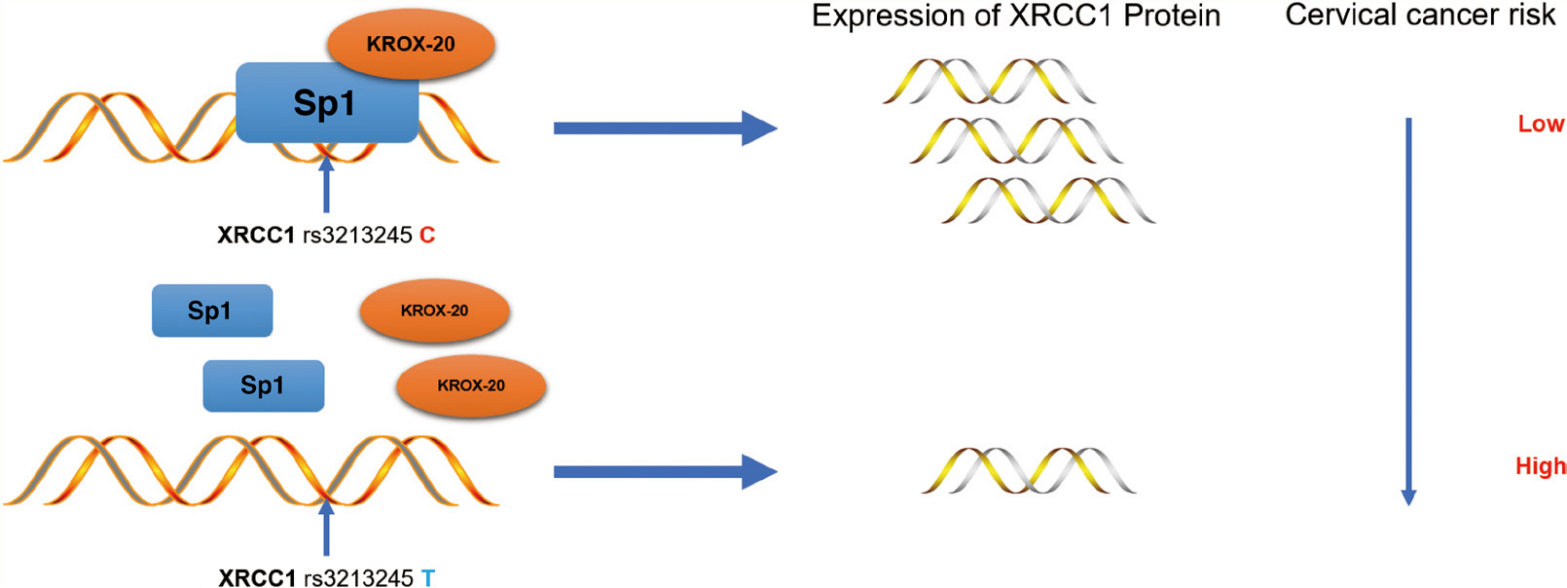
Schematic model of the regulations among rs3213245 and XRCC1 involved in cervical cancer development The transcription factor Krox-20 was recruited to the Sp1 binding motif. The rs3213245 C>T polymorphism may change the binding affinity of the transcription factor Sp1-Krox-20 complex to the mutation region, thereby regulating the expression of the *XRCC1* gene and ultimately leads to cervical cancer development.

Results also showed that the *XRCC1* rs3213245 C genotype decreases the risk of cervical cancer by upregulating the tumoral *XRCC1* transcription in cervical cancer patients. The ChIP and re-ChIP assays also demonstrated that the Sp1-Krox-20 complex prefers binding to the *XRCC1* rs3213245 C genotype, thereby enhancing its transcription (Figure 3).

In the present study, we found that common polymorphisms of *HOGG1, ADPRT* and *APE1* genes were not associated with the risk of cervical cancer. Moreover, *ADPRT* polymorphism was reported to be significantly associated with kinds of cancers [19, 20]. In the present study, the results of ADPRT rs1136410 genotypes associated with the risk of cervical cancer in two independent retrospective cohorts were different. It suggested that the polymorphisms of BER genes might be cellular-dependent because of different racial background [21]. Gene-environment interaction might play important role in the risk of cervical cancer. Larger studies should be taken to validate the association between ADPRT rs1136410 polymorphism and the risk of cervical cancer.

Further siRNA knockdown experiments showed that Krox-20 was recruited to Sp1 binding motif in both HeLa (HPV18–positive), SiHa (HPV16–positive), and C–33A (HPV–negative) cervical cancer cells *in vitro* knockdown experiments. This also confirmed that HPVs infection is not a necessary Etiological Factor for cervical cancer proposed by Wu et al. [22].

In conclusion, we have shown that the transcription factor Krox-20 was recruited to the XRCC1 rs3213245 mutation region and regulated the transcription of the *XRCC1* gene by interacting with Sp1, ultimately mediated cervical cancer development.

## MATERIALS AND METHODS

### Study subjects

This study comprised two independent retrospective cohorts. The testing cohort involved 571 cervical cancer patients and 657 matched cancer-free controls recruited from the First Affiliated Hospital of Nanjing Medical University between January 2007 and December 2010. The subjects in the validation cohort were recruited from hospitals in Nantong City between January 2009 and August 2016. This group comprised 608 cervical cancer patients and 1165 matched cancer-free controls. All included cases were newly and histopathologically diagnosed with primary cervical cancer. None of the patients had received chemotherapy or radiotherapy prior to enrolment. Each of the subjects provided informed consent. The experimental procedures were authorized by the ethics review board of the Southeast University.

### Genotype analysis

The functional genetic polymorphisms of the key components (i.e., *HOGG1, XRCC1, ADPRT*, and *APE1*) in the BER pathway were detected. The genomic DNA of the subjects in the testing cohort was extracted from the peripheral blood lymphocytes. The DNA of the validation cohort was extracted from the paraffin-embedded tissues. The polymorphisms of the selected genes were genotyped by the TaqMan allelic discrimination method by using the ABI 7900HT real-time PCR system (Applied Biosystems, CA, USA). The following primers were used to amplify the target fragments: rs1136410F5′-GACTGTAGGCCACCTCGATGTC-3′/R5′-AGTCTGTCTCATTCACCATGATACCT-3′, rs3213245F5′-TCTGGAGAGGCGCGACTG-3′/R5′-CAGAAGGATGAGGTAGAGTATGGG-3′, rs1799782F5′-GAGGATGAGAGCGCCAACTC-3′/R5′-TCACTCAGGACCCACGTTGTC-3′, rs 25489F5′-ATCTACTCTTTGTCTTCTCCAGTGC-3′/R5′-CTTCTCCTCGGGGTTTGCC-3′, rs25487F5′-AAGGAGTGGGTGCTGGACTGT-3′/R5′-CCAGCACAGGATAAGGAGCAG-3′, rs1760944F5′-AGCCTTCTCCACTGTTTTTTTCC/R5′-CAGCACATTGTGTGACACTGACTT-3′, rs1130409F5′-CCCGGCCTTCCTGATCAT-3′/R5′-CCCACCTCTTGATTGCTTTCC-3′, and rs1052133F5′-CCTCCTACAGGTGCTGTTCAGTG-3′/R5′-ACCCTTTCTGCGCTTTGCT-3′. Three positive control and negative control samples were used for the quality control of each amplification. At least 10% of the samples were randomly selected for further confirmation. The results were 100% concordant.

### Immunohistochemistry (IHC)

Tissues obtained from the cervical cancer patients were coated on 0.4 μm sections. IHC analysis was conducted as previously reported [23]. The sections were incubated with mouse XRCC1 (1:100 dilution, BOSTER) monoclonal antibodies incubated overnight at 4°C. XRCC1 staining in the sections was independently evaluated by two pathologists by using a semi-quantitative immunoreactivity score [24].

### Cell culture

HeLa, SiHa, and C–33A cells were cultured at 37°C in complete DMEM media (HyClone, Logan, UT, USA) with 10% FBS (Sigma, ST. Louis, MO, USA) and penicillin (100 U ml^-1^)/streptomycin (100 ug ml^-1^) (HyClone, Logan, UT, USA) in 5% CO2. The cells were periodically tested and validated to be mycoplasma free. For siRNA transfection experiments, three groups were separately transfected using Lipofectamine 2000 reagent (Invitrogen, Carlsbad, CA, USA), with 1μg negative control, Sp1, and Krox-20 siRNA (Genepharma, Shanghai, China) in accordance with the manufacturer’s protocol. Cells were collected 48h after transfection.

### Western blot (WB)

Total protein were extracted from cell samples as described before [25]. Proteins of cells were analyzed by immunoblots. Three primary antibodies were employed in the experiments: human Sp1 (1:1000 dilution; CST, USA), Krox-20(1:1000 dilution; abcam, USA), and α-tubulin (1:1000 dilution; Sigma, USA).

### Chromatin immunoprecipitation (ChIP) and Re-ChIP assays

Human peripheral white blood cells from the cervical cancer patients were used for the ChIP and Re-ChIP analyses according to the standard protocol [26]. Briefly, the antibody against Sp1, Krox-20, or control IgG were immunoprecipitated with the chromatin fragments in ChIP assay. For re-ChIP, the samples were diluted 10 times with the dilution buffer and subjected to the ChIP procedure again after the elution of the first ChIP. The chromatin–Sp1complex was re-immunoprecipitated using anti-human Krox-20, whereas the chromatin–Krox-20 complex was re-immunoprecipitated using anti-human Sp1. Quantitative PCR was performed to analyze the precipitated genomic DNA using the following human XRCC1 rs3213245 (-77TC) promoter primers: 5′-AGGAAACGCTCGTTGCTAAG-3′ (forward) and 5′-TGGCCAGAAGGATGAGGTAG-3′ (reverse) for rs3213245.

### Reverse transcription–quantitative real-time PCR (RT-PCR)

Total RNAs were extracted from frozen tissue samples of patients with cervical cancer using Trizol reagent (Invitrogen, US) and treated by DNase / RNase-free Deionized water (Tiangen, Beijing, China). cycA was used as internal controls in SYBR^®^ Green Realtime PCR Master Mix-Plus kits (Toyobo, Osaka, Japan). The expression level of XRCC1 were amplified with PCR primers (Takara, Shanghai, China) and quantitative using the Quant Studio 6 Flex system (Applied Biosystems, Life Technologies, USA).

### Statistical analysis

The χ^2^ test was used to investigate the Hardy–Weinberg equilibrium of the controls’ genotype frequencies and the frequency distribution of the selected demographic variables and genotypes. Multivariate logistic regression was used to calculate the odds ratio values and 95% confidence interval. *P* < 0.05 was considered statistically significant.

## SUPPLEMENTARY MATERIALS FIGURE


