# Supplementary material for: XRCC1 mediated the development of cervival cancer through a novel Sp1/Krox-20 swich
Source: Oncotarget. 2017 Sep 16;8(49):86217–26. doi: 10.18632/oncotarget.21040 (PMC5689679; doi:10.18632/oncotarget.21040)
Supplement: Supplementary file 1 [file oncotarget-08-86217-s001.pdf]

XRCC1 mediated the development of cervival cancer through a novel Sp1/Krox-20 swich

SUPPLEMENTARY MATERIALS

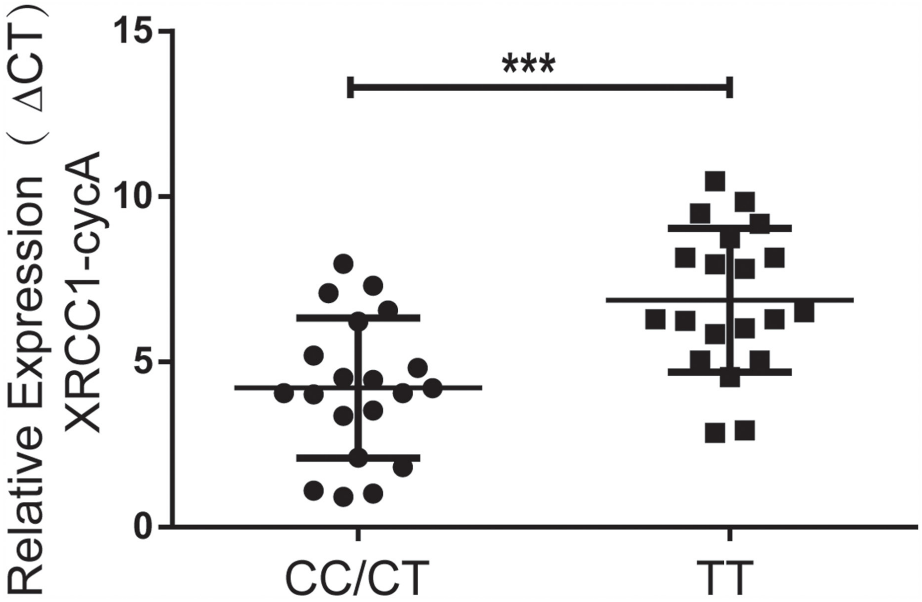

Supplementary Figure 1: Expression of XRCC1 were detected in cervical cancer patients carrying with the XRCC1 rs3213245 genotypes. ***: P<0.0001.
